# Papulonodular Mucinosis Associated With Subacute Lupus

**DOI:** 10.7759/cureus.23412

**Published:** 2022-03-23

**Authors:** Sabrine Rabba, Fouzia Hali, Farida Marnissi, Soumiya Chiheb

**Affiliations:** 1 Department of Dermatology, Ibn Rochd University Hospital, Casablanca, MAR; 2 Department of Pathology, Ibn Rochd University Hospital, Casablanca, MAR

**Keywords:** cutaneous lupus erythematosus, systemic lupus erythromatosus, subacute cutaneous lupus erythematosus, papulonodular mucinosis, mucinosis

## Abstract

Papulonodular mucinosis is a rare but well-documented finding associated with systemic and cutaneous forms of lupus erythematosus (LE). It occurs exceptionally in association with subacute cutaneous lupus erythematosus (SCLE). Its etiology and pathogenesis remain to be elucidated. Herein, we report a case of papulonodular mucinosis associated with SCLE in a middle-aged woman. On physical examination, she presented with multiple flesh-coloured asymptomatic papules and nodules on the trunk and upper extremities. A biopsy specimen taken from a nodule showed mucin within the dermis with hypodermis and perivascular lymphocytic inflammation. Considering that the proportion of patients with cutaneous lupus mucinosis who progress to systemic lupus is uncertain, we suggest following these patients closely for evidence of multisystem disease.

## Introduction

Mucin deposition is a common histopathologic finding in lupus erythematosus (LE) but is rarely sufficient to produce clinically apparent skin lesions [1]. The incidence of papulonodular mucinosis in patients with LE is 1.5% [2]. Herein, we describe a middle-aged woman with subacute cutaneous LE (SCLE) who subsequently developed papulonodular mucinosis.

## Case presentation

**Figure 1:**
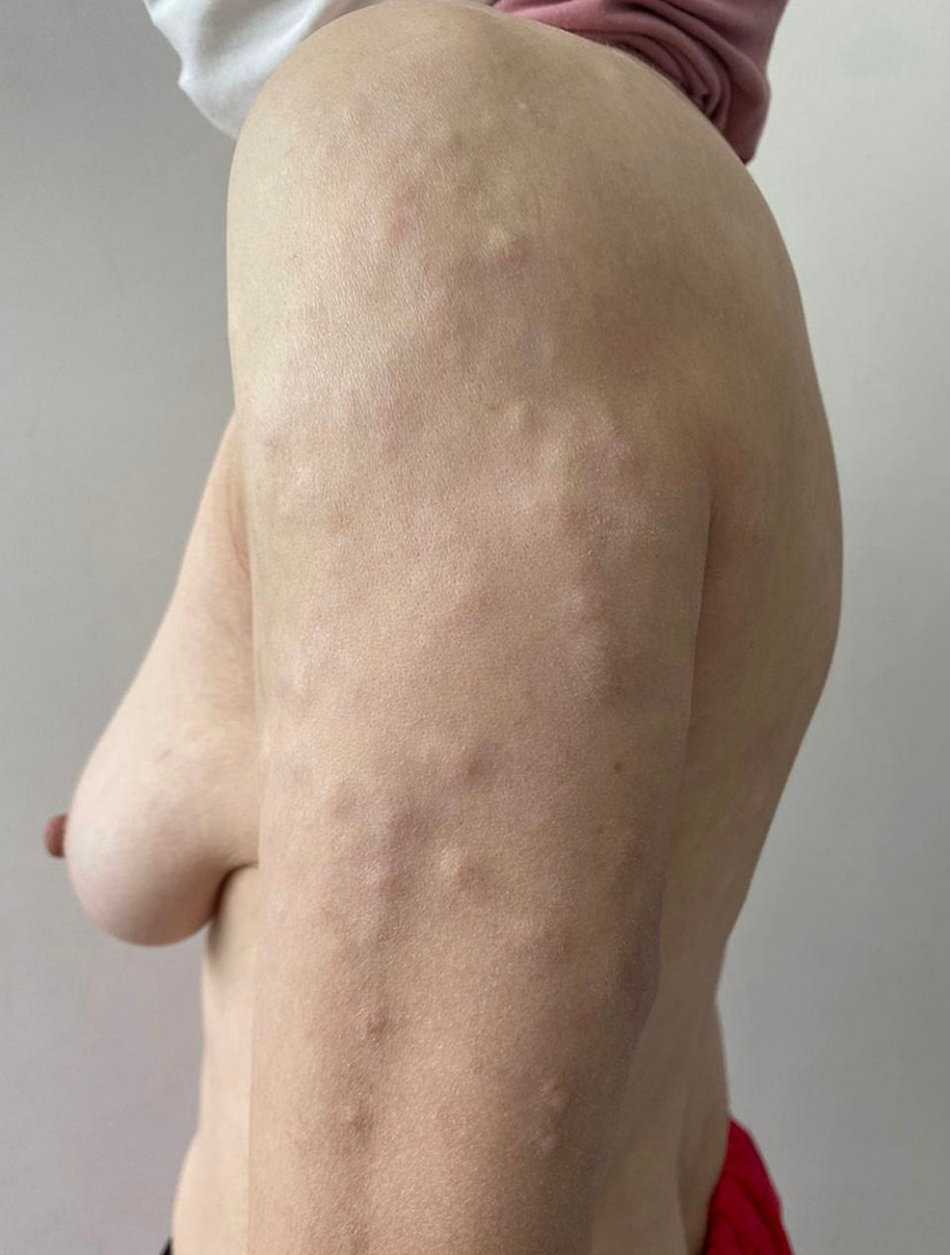
Flesh-coloured soft papules and nodules (upper limbs)

**Figure 2:**
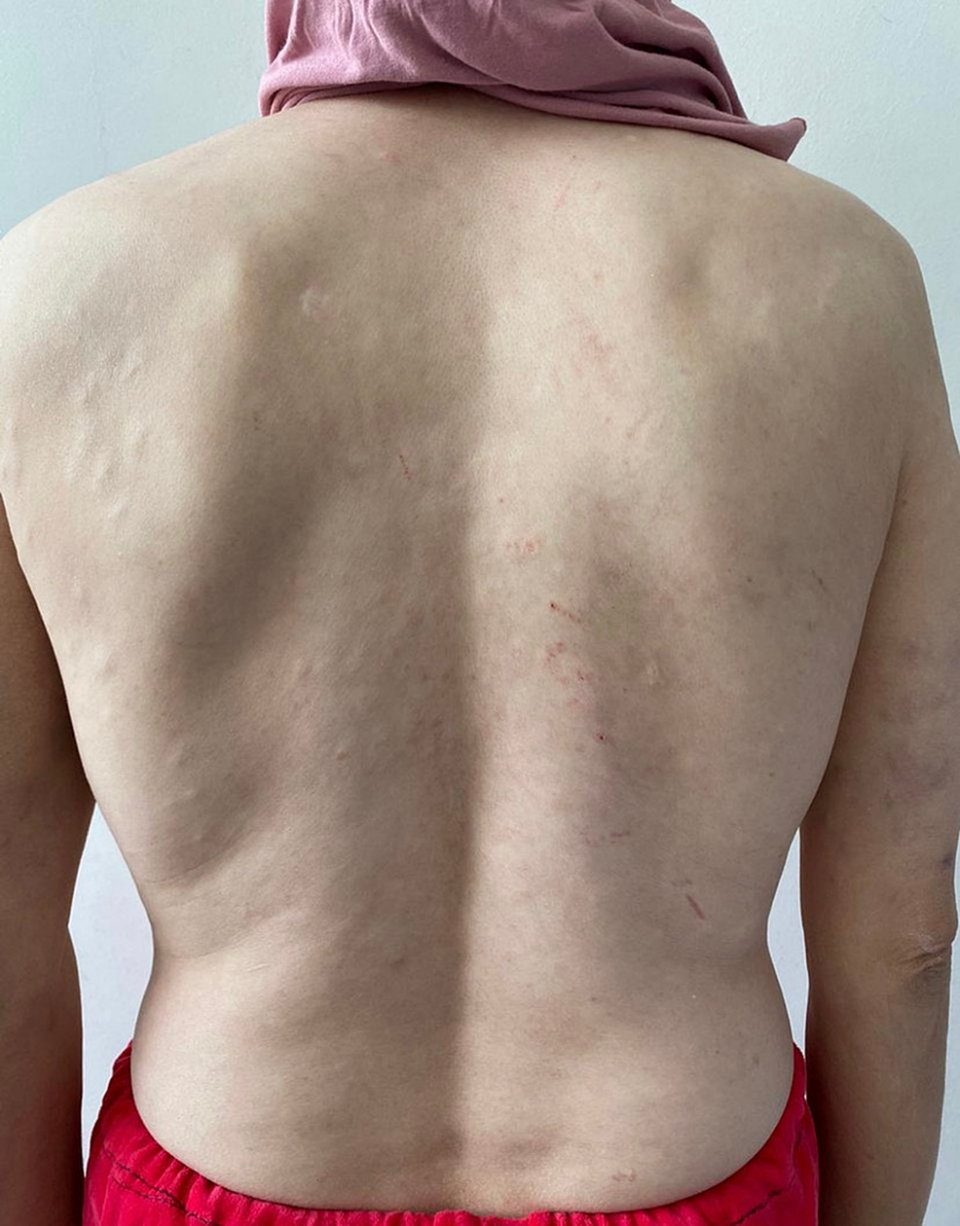
Flesh-coloured soft papules and nodules (trunk)

The patient, a 41-year-old woman, has been seen for nine years for skin lesions on her face, neck and scalp, consisting of erythematous, papulosquamous plaques and alopecia. SCLE was confirmed by histopathologic examination. She was treated with antimalarian agents and oral steroids with progressive degression. In the past months, multiple asymptomatic lesions had developped of the trunk and upper extremities. Therefore, she was referred to our dermatological outpatient department. On physical examination, the patient had multiple flesh-colored soft papules and nodules on the trunk and upper limbs (Figures 1, 2).

**Figure 3:**
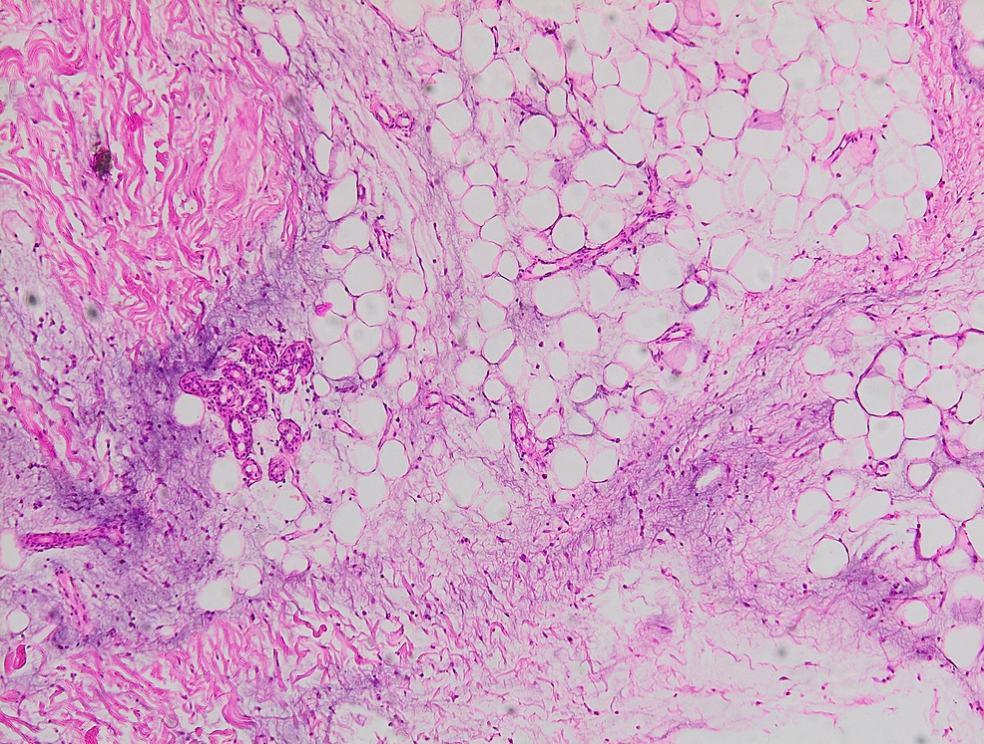
Dermic and hypodermic mucin deposition between collagen bundles (hematoxylin and eosin, original magnification x 100)

In addition to that, there were inherent LE lesions that were partially resolved. Histology showed a fibrotic dermis with profuse dermic and hypodermic mucin deposition between collagen bundles and perivascular lymphocytic inflammation (Figure 3).

Laboratory tests were positive for antinuclear and anti-dsDNA antibodies, but negative for Ro/SSA, La/SSB, U1RNP, Sm, and Scl-70. Further laboratory investigations, such as a complete blood cell count, kidney and liver function tests, electrolytes, and complement components, were within normal limits, and physical examination revealed no systemic involvement. We added topical steroids to the treatment and increased the dose of antimalarian agents (chloroquine phosphate 4mg/kg per day); the lesions flattened within three months.

## Discussion

Gold was the first to describe an unusual papulonodular mucinosis associated with LE in 1954 [3]. This uncommon entity is a primary cutaneous mucinosis that has been generally described in systemic lupus erythematosus, but also in discoid LE, and rarely SCLE. To date, 53 cases have been documented in English literature. Among them, only three cases have been associated with SCLE. In some patients, it predates LE and in others it is associated with its activity. It can even sometimes be the only cutaneous manifestation of LE [4].

This entity has an unclear pathogenesis. One study has postulated that the production of glycosaminoglycans by dermal fibroblasts is increased and thought to be stimulated by a factor (or factors) in the patient's serum that is as yet unidentified [5]. It is documented that ultraviolet light exposure can aggravate these lesions [1]. Despite a higher incidence of SLE in women, lupus cutaneous mucinosis occurs more frequently in men. This indicates a possible role for sex-related factors, such as androgenic hormones, in the pathogenesis of this associated disease [4].

Typically, papulonodular mucinosis appears as asymptomatic, flesh-colored papules and nodules that can be seen inside and outside of typical lesions of LE with a propensity for the trunk and upper extremities [2]. Exposure to sunlight might be a further contributing factor in the pathogenesis of this disease [1]. The histologic picture is dominated by mucin deposits that are dispersed throughout the dermis, interspersed within collagen bundles. What is distinctive of the condition is the lack of the histological changes characteristic of other lupus erythematosus eruptions such as epidermal involvement and vacuolar degeneration of the dermoepidermal junction [2,4]. Direct immunofluorescence may demonstrate linear or granular deposits of immunoglobulin and/or complement protein [2].

Treatment with glucocorticosteroids (topical, intralesional, or systemic), antimalarial agents, retinoids, cyclophosphamide, methotrexate, plasmapheresis or surgical options, such as dermabrasion, laser, or excision, have been shown to be of benefit sometimes. For refractory cases, hyaluronidase and triamcinolone injections have been employed successfully [2,4,6].

## Conclusions

Papulonodular mucinosis is a rare manifestation of different subtypes of LE, but is most frequently reported for patients with systemic LE. Considering that the proportion of patients with cutaneous lupus mucinosis who progress to systemic lupus is uncertain and the rarity of this condition, we suggest following these patients closely for evidence of multisystem disease. 
